# Dual Oxygen and Temperature Luminescence Learning Sensor with Parallel Inference

**DOI:** 10.3390/s20174886

**Published:** 2020-08-28

**Authors:** Francesca Venturini, Umberto Michelucci, Michael Baumgartner

**Affiliations:** 1 Institute of Applied Mathematics and Physics, Zurich University of Applied Sciences, Technikumstrasse 9, 8401 Winterthur, Switzerland; Michael.Baumgartner@zhaw.ch; 2 TOELT LLC, Birchlenstrasse 25, 8600 Dübendorf, Switzerland; umberto.michelucci@toelt.ai; 3 School of Computing, University of Portsmouth, Portsmouth PO1 3HE, UK

**Keywords:** artificial intelligence, neural network, machine learning, oxygen sensor, luminescence, optical sensor, luminescence quenching, phase fluorimetry

## Abstract

A well-known approach to the optical measure of oxygen is based on the quenching of luminescence by molecular oxygen. The main challenge for this measuring method is the determination of an accurate mathematical model for the sensor response. The reason is the dependence of the sensor signal from multiple parameters (like oxygen concentration and temperature), which are cross interfering in a sensor-specific way. The common solution is to measure the different parameters separately, for example, with different sensors. Then, an approximate model is developed where these effects are parametrized ad hoc. In this work, we describe a new approach for the development of a learning sensor with parallel inference that overcomes all these difficulties. With this approach we show how to generate automatically and autonomously a very large dataset of measurements and how to use it for the training of the proposed neural-network-based signal processing. Furthermore, we demonstrate how the sensor exploits the cross-sensitivity of multiple parameters to extract them from a single set of optical measurements without any a priori mathematical model with unprecedented accuracy. Finally, we propose a completely new metric to characterize the performance of neural-network-based sensors, the Error Limited Accuracy. In general, the methods described here are not limited to oxygen and temperature sensing. They can be similarly applied for the sensing with multiple luminophores, whenever the underlying mathematical model is not known or too complex.

## 1. Introduction

The simultaneous determination of multiple physical quantities can be very advantageous in many sensor applications, for example, when an in-situ or a remote acquisition is required. If the physical effect on which the measurement method is based presents cross-sensitivity between more than one quantity, their simultaneous determination becomes a necessity. Optical luminescence sensing is particularly attractive for multiple sensing. Since several parameters can be measured using the same principle, namely luminescence, it is possible to use the same illumination or detection channels, thus allowing a compact and simple sensor design.

The typical approaches to multiple sensing are based on either the use of a single luminescence indicator (luminophore), in which the luminescence is sensitive to more than one physical quantity, or the use of several luminophores, one for each quantity, embedded in a substrate and placed in close physical proximity [1,2,3,4,5,6,7,8,9]. To be able to determine each quantity separately, it may be necessary to determine more than one optical property (e.g., absorption spectrum, emission spectrum, luminescence intensity, decay time). Another possibility is to measure one single optical property using special detection schemes that take advantage of the emission properties of the used luminophores [4,6,10,11,12,13].

The problem of dual sensing is particularly relevant in applications that involve oxygen sensing. Since oxygen plays a major role for living organisms, the measurement of oxygen partial pressure is of great relevance in fields which range from medicine and biotechnology, to environmental monitoring [4,14]. One of the most used optical measuring approaches is based on dynamical luminescence quenching. When colliding with molecular oxygen, the energy of the excited luminophore is reduced due to radiationless deactivation. As a result, both the intensity and decay time of the luminescence are reduced (quenched) [15]. The dependence of the measured sensing quantity (e.g., luminescence intensity or decay time) on the relevant influencing factors needs to be described through mathematical models with a sufficiently complex parametrization. Among the cross-interfering quantities, temperature is the most relevant since both the luminescence and its quenching are strongly temperature-dependent phenomena. Therefore, in any optical oxygen sensor, the temperature must be continuously monitored, most frequently with a separate sensor, and used to correct the calculated oxygen concentration [16]. This task can be difficult in practical implementation and may become a significant source of error. Another disadvantage of this approach is that the parametrization of the sensor response is system-specific since it depends on how the sensing element was fabricated and on the sensor itself [17,18,19,20,21,22].

In this work, these difficulties are overcome through a new approach for sensor development based on neural networks for parallel inference. This enables accurate dual-sensing, using one single luminophore and by measuring a single quantity. Instead of describing the response of the sensor as a function of the relevant parameters through an analytical model, a neural network was designed and trained to predict both oxygen concentration and temperature simultaneously. Multi-task learning (MTL) architectures were chosen for this new approach because they can learn correlated tasks [23,24,25,26,27,28]. In a previous purely theoretical study that used only synthetic data, the authors showed that MTL architectures can be flexible enough to address multi-dimensional regressions problems [29] as required by this new type of sensor. This work demonstrates for the first time that this is indeed true by building, training and characterizing a real physical optical sensor based on this principle. To train the MTL neural network and to test the performance of the sensor on unseen data, a very large amount of data is needed. Since the collection cannot be performed by hand, a fully automated data collection setup was developed and used to both vary the sensor environment conditions (gas concentration and temperature) and to collect the sensor response.

This work proposes a paradigm shift from the classical description of the response of a sensor through an approximate model, to the use of MTL based sensor learning thanks to neural networks. These will learn the complex inter-parameter dependencies and sensor-specific response characteristics from a large amount of data automatically collected. This new method will enable to build sensors even if the response of the system to the physical quantities is too complex to be comfortably described by a mathematical model.

## 2. Methods

### 2.1. Measurement Principle

Luminescence-based oxygen sensors usually are based on a luminophore in which luminescence intensity and decay time decrease for increasing O_2_ concentrations. This reduction is due to collisions of the excited luminophore with molecular oxygen, which thus provides a radiationless deactivation process. The dependence of the luminescence intensity and decay time of the luminophores used for oxygen sensing is best described by the Stern–Volmer (SV) equation [15]. Using a frequency-domain approach, the phase shift difference between the excitation and the emitted luminescence can be approximated using a two-site model [30,31] and written as [32]

(1) $$\begin{array}{cc} \frac{\tan\theta_{0}\left(\omega ,T\right)}{\tan\theta (\omega ,T,\left[O_{2}\right])}=\; & (\frac{f(\omega ,T)}{1+K_{SV1}\left(\omega ,T\right)\cdot \left[O_{2}\right]}+\\ \; & \frac{1-f(\omega ,T)}{1+K_{SV2}\left(\omega ,T\right)\cdot \left[O_{2}\right]}{)}^{-1} \end{array}$$

where $\theta_{0}$ and θ, are the phase shifts without and with oxygen, *f* and 1−f are the fractions of the total emission for the two components, $K_{SV1}$ and $K_{SV2}$ are the corresponding Stern–Volmer constants, and ω is the angular modulation frequency. It is to be noted that the quantities $\theta_{0}$, *f*, $K_{SV1}$, and $K_{SV2}$ are all temperature dependent [33,34,35]. Additionally, they also depend on the modulation frequency, which in the case of *f*, $K_{SV1}$, and $K_{SV2}$ is an artifact due to the approximate nature of the model. Finally, Equation (1) needs to be inverted to determine $\left[O_{2}\right]$ from the measured quantity θ.

From Equation (1) it is evident that the phase shift cannot be easily used to determine the oxygen concentration unless ω and *T*, the parameters *f*, $K_{SV1}$ and $K_{SV2}$ (including their dependencies from ω and *T*) are known. The proposed sensor not only overcomes the above-mentioned difficulties in finding an approximate mathematical model, but also allows the determination of multiple quantities simultaneously.

### 2.2. Experimental Setup and Dataset

**Figure 1:**
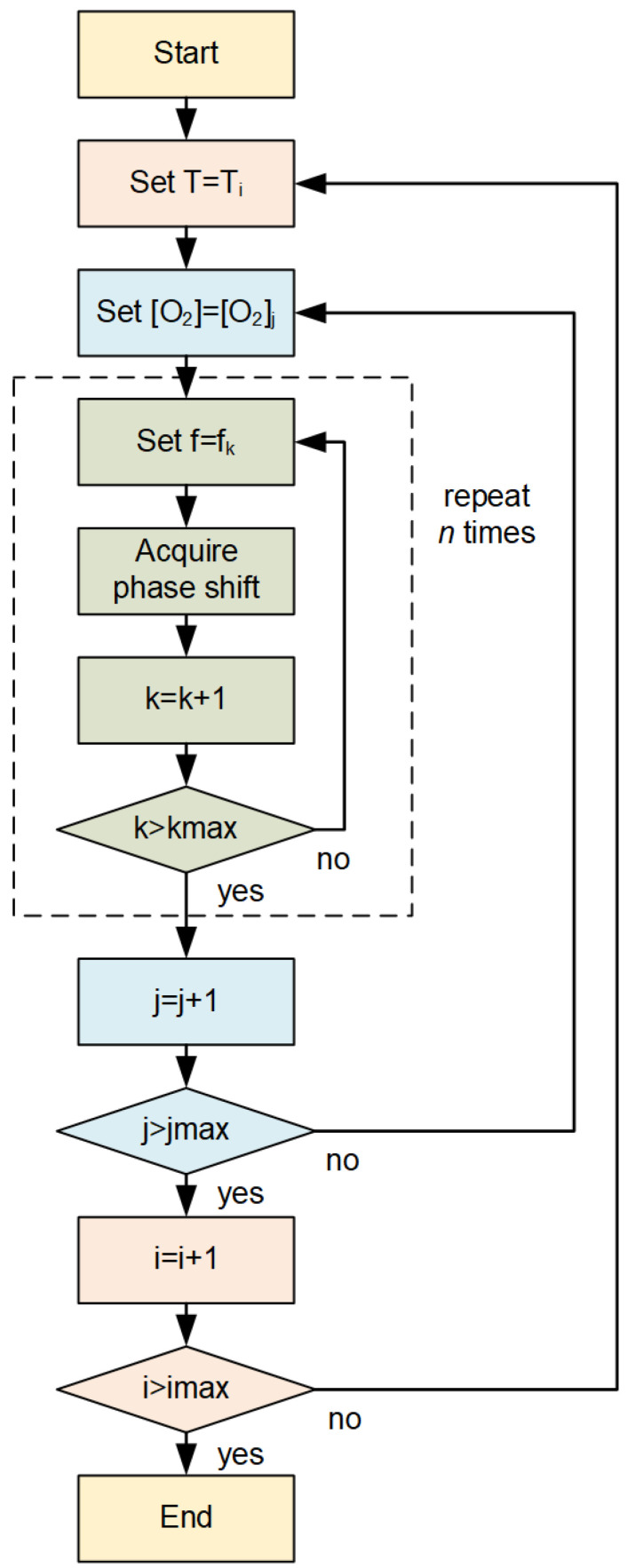
Flow-chart of the automated data acquisition program.

The luminophore used for oxygen detection is Pt-TFPP, commercially available as Oxygen Sensor Spot (PSt3, PreSens GmbH, Regensburg, Germany). The optical setup for the luminescence measurements is described in [36]. The large amount of data needed for the training and the test of the neural network was acquired using an automated acquisition program written using the software LabVIEW by National Instruments. The flow chart of the automated data acquisition program is shown in Figure 1.

First, the program fixed the temperature and concentration of the gas in contact with the sensor. Then, the phase shift was measured for 50 modulation frequencies between 200 Hz and 15 kHz. This measurement was repeated 20 times. Next, keeping the temperature fixed, the program changed the oxygen concentration, and the entire frequency-loop was repeated. The oxygen concentration was varied between 0% air and 100% air in 5% air steps. Finally, the temperature was changed, and then the oxygen and frequency loops where repeated. The temperature was varied between 5 °C and 45 °C in 5 °C steps. The total number of measurements was thus 50 (frequencies) times 20 (loops) times 21 (oxygen concentrations) times 9 (temperatures) for a total of 189’000, which required a total acquisition time of approximately 65 h. This number of measurements was chosen as a compromise between maximizing the number of data and avoiding photodegradation, which naturally occurs when the sample is subjected to illumination.

### 2.3. Signal Processing Algorithm

The software component of this new sensor type is based on a neural network model (NNM). An NNM is made of three components [37]: a neural network architecture (that includes how neurons are connected, the activation functions and all the hyperparameters), a loss function (here indicated with *L*) and an optimizer algorithm. In this particular work we use what is called a Multi Task Learning (MTL) network architecture [25]. This architecture has different branches, each able to learn to predict a separate quantity (in our case one *T* and [$O_{2}$]). The details and parameters of the neural network architecture, of the loss function and of the optimizer used in this work are studied and described in detail in [29] and will not be described again here.

The network was trained with two types of input to test its effectiveness. In the first case, each observation consists of a vector of 50 values defined as

(2) $$\theta_{s}=\left(\frac{\theta (w_{1})}{90},\frac{\theta (w_{2})}{90},...,\frac{\theta (w_{50})}{90}\right)$$

where $w_{i}$ are the 50 values of the angular modulation frequency of the excitation light (see Section 2.2). The measured phase shift was divided by 90 to normalize the inputs between 0 and 1. In the second case, each observation is

(3) $$\theta_{n}=\left(\frac{\theta (w_{1})}{\theta_{0}\left(w_{1}\right)},\frac{\theta (w_{2})}{\theta_{0}\left(w_{2}\right)},\ldots ,\frac{\theta (w_{50})}{\theta_{0}\left(w_{50}\right)}\right)$$

where $\theta_{0}\left(w_{i}\right)$ is the value of the measured phase shift without oxygen quenching at the angular modulation frequency $w_{i}$.

The loss function was minimized using the optimizer Adaptive Moment Estimation (Adam) [37,38]. The implementation was performed using the TensorFlow™ library. The training was performed with a starting learning rate of $10^{-3}$. Two types of training were investigated to compare the training efficiency and performance of the network. *No-batch training*: with this method all the training data are used to perform an update of the weights and to evaluate the loss function. *Mini-batch training*: with this method the weights update is performed after the network has seen 32 observations (this number is called mini-batch size [37]). For each update of the weights, 32 random observations are chosen from the training dataset without repetitions until all the training data are fed to the network. The size of the mini-batch was chosen as a compromise between a good performance (measured through the value of the loss function) and the duration of training.

No-batch training has the advantage of stability and requires less time for each epoch since it performs one update of the weights using the entire training dataset. Mini-batch training is normally more effective in reaching small values of the loss function in fewer epochs, but it requires more time for each epoch [37]. In our experiments the training for 20’000 epochs took roughly five minutes for no-batch training, and approximately 1 h with mini-batch training with mini-batch size of 32, thus resulting ca. 12 times slower. The training has been performed on a 2.2 GHz 6-Core Intel Core i7, with 32 GB of RAM. No GPU acceleration was used.

### 2.4. Sensor Performance Evaluation

To evaluate the performance of the sensor, the dataset *S* of measured data was divided into two parts: one containing 80% of randomly chosen observations (indicated with $S_{train}$), and one containing the remaining 20% of the data (indicated with $S_{test}$). All the results presented here were obtained by measuring the different metrics on the $S_{test}$ dataset.

The metric used to compare predictions from expected values is the absolute error (AE) defined as the absolute value of the difference between the predicted and the expected value for a given observation. The mean of the AE overall observations of a given dataset is the mean absolute error MAE and is a further metric used to characterize the performance. In Section 3, the prediction distribution of the AEs for both the oxygen and temperature predictions is discussed in detail. To better illustrate this distribution, the kernel density estimate (KDE) of the AEs was also evaluated. Details on the calculation of the AE, MAE and KDE can be found in [29].

#### Error Limited Accuracy

Generally, in a commercial sensor, the accuracy quantifies the performance of the sensor and helps to decide if the chosen device is appropriate for the application of interest. The above-defined metrics (AE, MAE and KDE) are useful to compare the performance of different NNMs but do not help quantify which error the sensor reading will ultimately have in practice. For this reason, in this work we introduce a new metric, called Error Limited Accuracy (ELA) and indicated with η.

**Definition** **1.** *In a regression problem, given the metric* AE *, and a chosen value of it* $\hat{AE}$ *, the* ELA *η limited by the error* $\hat{AE}$ *is defined as the number of predictions* $\hat{y}$ *of the NNM that lie in the range* $|\hat{y}-y|\leq \hat{AE}$ *, with y the expected value, divided by the total number of observations. It will be indicated with* $\eta (\hat{AE})$ *. Given the set*

(4) $$E\left(\hat{AE}\right)=\{{\hat{y}}^{\left[i\right]}\;with\;i=1,...,n\;|\;\;|{\hat{y}}^{\left[i\right]}-y^{\left[i\right]}\left|\leq \hat{AE}\right\}$$

$\eta (\hat{AE})$ *is defined as*

(5) $$\eta \left(\hat{AE}\right)=\frac{\left|E(\hat{AE})\right|}{n}$$

*where* $\left|E(\hat{AE})\right|$ *is the cardinality of the set* $E(\hat{AE})$ *or, in other words, the number of its elements.* $y^{\left[i\right]}$ *and* ${\hat{y}}^{\left[i\right]}$ *are respectively the expected and predicted value of the target variable for observation i.*

This metric allows interpreting the regression problem as a classification one. $\eta (\hat{AE})$ simply describes how many observations are predicted by the NNM within a given value of the absolute error. In other words, it represents the percentage of predictions that are within a certain error $\hat{AE}$. Therefore, if we make $\hat{AE}$ big enough, all the predictions will be classified perfectly, so $\eta (\hat{AE})$ is expected to approach 1 for increasing $\hat{AE}$. On the other hand, the smaller $\hat{AE}$ is, the lower will be the number of predictions correctly classified. We finally define $\bar{AE}$ as the minimum value of $\hat{AE}$ for which $\eta (\hat{AE})=1$, so the minimum value of the absolute error for which the network predicts all the observations correctly. This value ($\bar{AE}$) can be interpreted as the biggest error in the sensor predictions.

## 3. Results and Discussion

### 3.1. Pt-TFPP Luminescence

**Figure 2:**
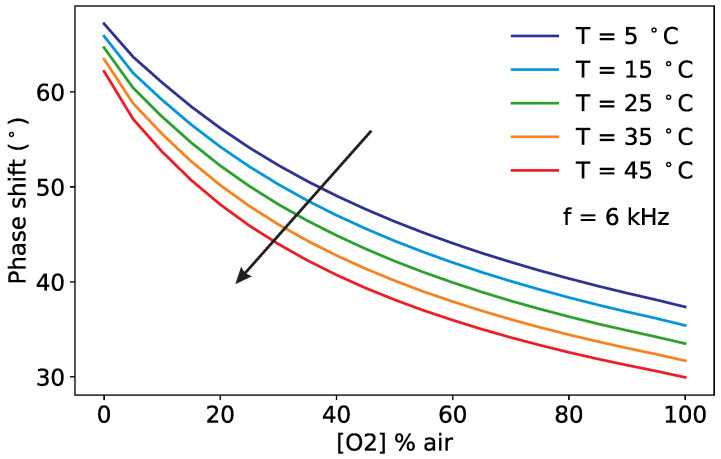
Measured phase shift as a function of the oxygen concentration for selected temperatures at a fixed modulation frequency of 6 kHz. The arrow marks increasing temperatures.

**Figure 3:**
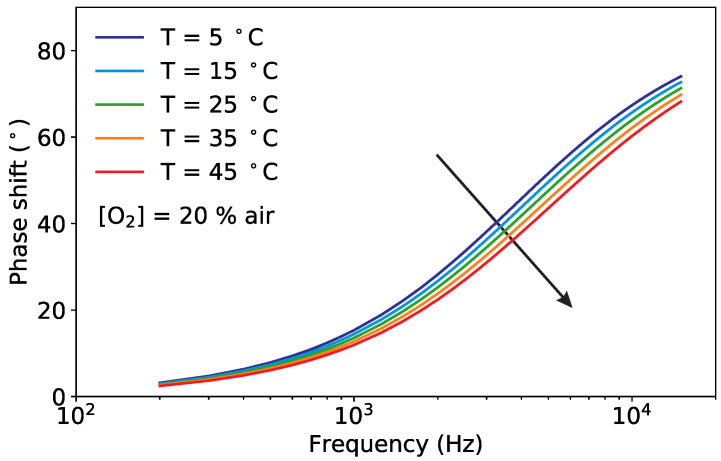
Measured phase shift as a function of the modulation frequency for selected temperatures at a fixed oxygen concentration of $[O_{2}]=20\%$ air. The arrow marks increasing temperatures.

**Figure 4:**
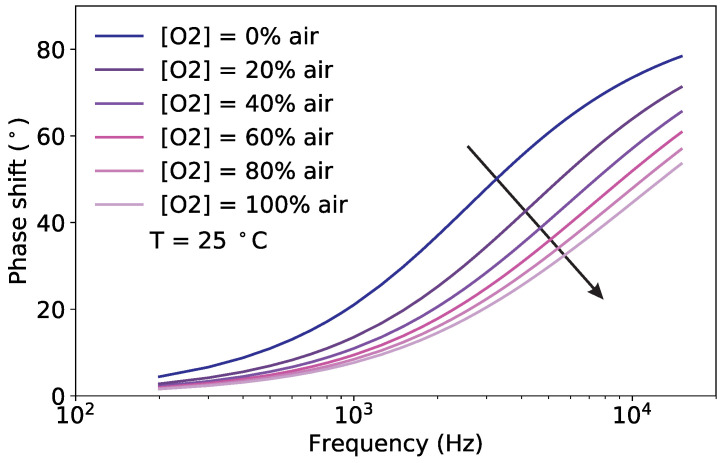
Measured phase shift as a function of the modulation frequency for selected oxygen concentrations at a fixed temperature of T=25 °C. The arrow marks increasing oxygen concentrations.

As described in Section 2.1, the phase shift depends non-linearly on the oxygen concentration according to the Stern–Volmer equation. It also depends on the temperature, which influences the luminescence and the collision mechanisms, and on the modulation frequency of the excitation light. The experimental observations for the phase shift for variations of these three quantities are shown in Figure 2, Figure 3 and Figure 4.

Figure 2 shows the measured phase shifts as a function of the oxygen concentration at a constant modulation frequency of 6 kHz and for increasing temperatures. For clarity, the results at selected temperatures are shown. The decrease of the phase shift due to the collisional quenching is clearly visible in all curves. The phase shift is, as expected, also strongly temperature-dependent. For [$O_{2}$] = 0, in the absence of oxygen, the reduction of the phase shift with increasing *T* is due to temperature quenching; the influence of temperature becomes stronger at higher oxygen concentration, as a result of the increase of the diffusion rates of oxygen through the sample.

For a given oxygen concentration, the phase shift is strongly dependent on the modulation frequency, as it can be seen in Figure 3, where the shape of the frequency response is determined by the distribution of decay times of the sample. From the figure it is visible that the reduction of the phase shift with increasing temperatures is not constant but depends on the modulation frequency.

For completeness, the effect of the oxygen concentration on the frequency response at a fixed temperature is shown in Figure 4. Compared to Figure 3, the frequency response of the sample is affected more strongly by the oxygen concentration than by temperature. In other words, the sample has a higher sensitivity to oxygen than to temperature.

The measurements of Figure 2, Figure 3 and Figure 4 show how similar the curves of the phase shift are for different values of oxygen, temperature and modulation frequency. This helps to understand why it is not possible from the measurement of the phase shift, or even of the phase shift for varying modulation frequencies, to simultaneously determine both the oxygen concentration and the temperature using Equation (1). The temperature must be known in advance and used to compute the oxygen concentration. This is no longer the case for the proposed sensor, as it will be shown in the next section.

### 3.2. Sensor Performance

**Figure 5:**
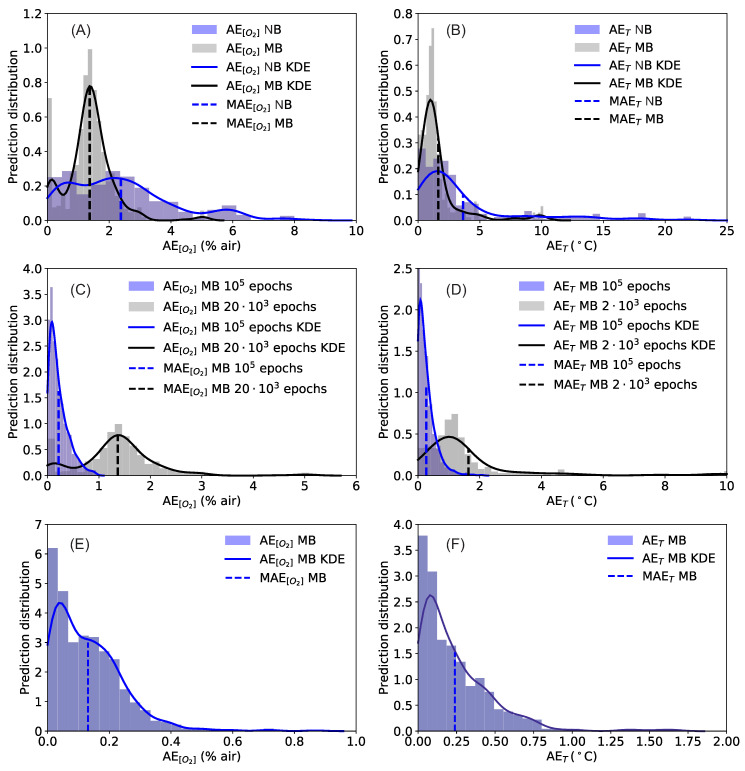
Distributions of the neural network predictions for the oxygen concentration (panels (**A**,**C**,**E**) and for the temperature (panels (**B**,**D**,**F**). In all panels the normalized prediction distribution histogram (columns), the kernel density estimate KDE of the distribution of the absolute errors AEs (solid line), and mean absolute errors MAEs (dashed vertical line) are shown. Panels (**A**,**B**): Comparison between training using no batches (NB) and using mini-batches (MB) with a batch size of 32 both trained for 20’000 epochs; the input of the network is $\theta_{s}$. Panels (**C**,**D**): Comparison between training using mini-batches (MB) with a batch size of 32 for 100’000 and 20’000 epochs; the input of the network is $\theta_{s}$. Panels (E) and (F): results with a training using mini-batches (MB) with a batch size of 32 for 20’000 epochs and using the input of the network is $\theta_{n}$.

First, the effect of the training on the sensor performance was investigated. As described previously, the neural network was trained with no-batches and with mini-batches. For this comparison the network was trained for 20’000 epochs using the input observations $\theta_{s}$ as defined in Equation (2). The results for $AE_{\left[O_{2}\right]}$ and $AE_{T}$ are shown in Figure 5A,B, respectively. The blue histogram shows the AE distribution when using no-batch, the gray when using mini-batches of size 32. The KDE profiles help to illustrate the features of the histogram. The effect of introducing mini-batches on the performance is significant. The predictions distributions get much narrower, the mean average errors decrease from $MAE_{\left[O_{2}\right]}=2.4$% air and $MAE_{T}=3.6$ °C to $MAE_{\left[O_{2}\right]}=1.4$% air and $MAE_{T}=1.6$ °C. Although the performance is significantly improved, from Figure 5A,B it can also be clearly seen that errors as high as approximately 5% air for $\left[O_{2}\right]$ or 12 °C for *T* are still possible.

Figure 5C,D shows the effect of the training length. Here the comparison is between prediction distributions with 20’000 and 100’000 epochs (always using a mini-batch of size 32), using the input observations $\theta_{s}$ as defined in Equation (2). The effect of longer training is a dramatic improvement in the performance. When the network was trained for 100’000 epochs the mean average errors were reduced to only $MAE_{\left[O_{2}\right]}=0.22$% air and $MAE_{T}=0.27$ °C. Additionally, all the predictions for $\left[O_{2}\right]$ lie below 0.94% air, and for *T* lie below 2.1 °C.

The results of Figure 5C,D demonstrate two new findings: (1) with the proposed approach, it is possible to predict both $\left[O_{2}\right]$ and *T* at the same time from the phase shift using a single luminophore and a set of measurements; (2) the prediction has an expected error that is comparable or below the typical accuracy of commercial sensors. The possibility of dual sensing paves the road to the development of a completely new generation of sensors. The price to pay is that the training of a network for 100’000 epochs requires approximately 5 h on the hardware described earlier.

To investigate if the training can be performed more efficiently, the normalized phase shift $\theta_{n}$ defined in Equation (3) was used as input to the network. The performance of the network in this case, with a mini-batch size of 32 and a training of 20’000 epochs is shown in Figure 5E,F. With this input the performance is further improved: even if the number of epochs is only 20’000 the mean average errors are better than what was obtained with $\theta_{s}$ and a training of 100’000 epochs, achieving $MAE_{\left[O_{2}\right]}=0.13$% air and $MAE_{T}=0.24$ °C. The distributions are also narrower, particularly for the temperature. Additionally, all the $AE_{\left[O_{2}\right]}$ lie below 0.87% air, and $AE_{T}$ below 1.7 °C. This type of training is clearly more efficient. The reason may lie in the additional information which is fed to the network when using the input $\theta_{n}$ and in the simplified functional behavior of $\theta_{n}$ compared to $\theta_{s}$ (see Equation (1)).

The performance of the different neural networks is summarized in Table 1.

The response time of the sensor is due to the sum of two contributions: the actual measurement time of the phase shift and the time needed by the algorithm to calculate the oxygen concentration and temperature. The measurement time for 50 frequencies with our setup was below one minute but could be easily improved by reducing the time delays in the communication between the various instruments.

### 3.3. Error Limited Accuracy

The metrics discussed in the previous sections are useful to compare the network performance and to measure how good the predictions are. However, they do not offer an understanding on what a sensor built with such a model could achieve. For practical applications, the relevant question is rather what is the maximum error the sensor will have in predicting the oxygen concentration and temperature. To answer this question, the ELA (η) defined in Section 2.4 can be used.

**Figure 6:**
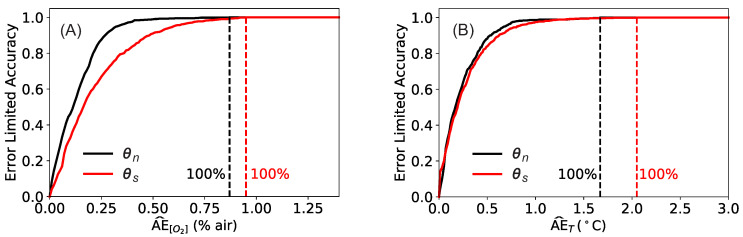
Comparison of the ELAη: Panel (**A**) oxygen prediction, panel (**B**) temperature prediction. The black lines are the results obtained with a network that was trained with $\theta_{n}$ as input for 20’000 epochs with mini-batchs of size 32, while the red ones with $\theta_{s}$ as input for 100’000 epochs with mini-batchs of size 32. The dashed lines indicates the values of the $\bar{AE}$ for which the predictions would give η=1.

Figure 6 displays the ELA$\eta (\hat{AE})$ for oxygen concentration (A) and for the temperature (B). In each panel, the results obtained with the bests models described before are shown: the ELAs using the input $\theta_{n}$ and a training for 20’000 epochs are shown in black, and the ELAs obtained using the input $\theta_{s}$ and a training for 100’000 epochs in red. In both cases, the training was performed with mini-batches of size 32. The dashed lines indicate the values of the ${\bar{AE}}_{\left[O_{2}\right]}$ and ${\bar{AE}}_{T}$ for which the error limited accuracy η equals 1. In other words, all the predictions will have an error equal or smaller than $\bar{AE}$.

From Figure 6A can be seen that, for the network trained with $\theta_{s}$ as input, the model would predict perfectly all the oxygen concentrations within 0.95% air error. For the network trained with $\theta_{n}$ this value is further reduced to 0.87% air. ${\bar{AE}}_{\left[O_{2}\right]}$ can be interpreted as the accuracy a sensor based on this NNM would have. Figure 6B shows the results of the same analysis for the temperature measurement. The interpretation is similar to the one given above for the oxygen concentration. For the network trained with $\theta_{s}$ as input, the model would predict perfectly all the temperature values within ${\bar{AE}}_{T}=2.1$ °C error. For the network trained with $\theta_{n}$ this value would be ${\bar{AE}}_{T}=1.7$ °C. The values of ${\bar{AE}}_{\left[O_{2}\right]}$ and ${\bar{AE}}_{T}$ are summarized in Table 2.

## 4. Conclusions

In this work, the realization of a new type of sensor based on luminescence sensing is presented. The proposed sensor allows parallel inference, or the extraction of multiple physical quantities simultaneously, from a single set of measurements without any a priori mathematical model, even in the presence of cross interferences. Classical approaches to this type of problem in physics can be challenging or impossible to solve if the mathematical models describing the functional dependencies are too complex or even unknown.

This sensor, which uses a single luminophore and a single measuring channel can measure simultaneously both the oxygen concentration and the temperature of a medium. This is achieved using a multi-task learning neural network model, which was trained on a very large dataset. The results in the prediction of the oxygen concentration and temperature show unprecedented accuracy for both parameters, demonstrating that this approach could open up the possibility of a new generation of dual- or even multiple-parameter sensors. Estimating the accuracy of a sensor based on a given NNM approach is intrinsically difficult. For this reason, the new metric Error Limited Accuracy ELA is proposed. The ELA enables to estimate how many predicted values lie within a certain absolute error from the expected measurement. This new metric allows therefore the estimation of the maximum measurement error of any NNM-based sensor.

The ability to predict both $\left[O_{2}\right]$ and *T* at the same time, from a single set of data obtained with a single indicator, has profound implications for the development of luminescence sensors. Sensors will become easier and cheaper to build since no separate temperature measurements are necessary anymore. Generally, this work shows that the effect of interferences can be learned by the neural network and do not need to be corrected for in the data processing.

This work opens the road to complete new optical sensing approaches for future generations of sensors. Those sensors will be able to extract multiple physical quantities from a common set of data at the same time to achieve consistent results that are both accurate and stable. The described approach is relevant for many practical applications in sensor science and demonstrates that this model-free approach has the potential of revolutionizing optical sensing.

## Figures and Tables

**Table 1:** Summary of the performance of the sensor for different neural network models.

Input	Epochs/Batch Size	${\mathrm{MAE}}_{\left[O_{2}\right]}$	${\mathrm{MAE}}_{\left[T\right]}$
$\theta_{s}$	20’000/no batch	2.4% air	3.6 °C
$\theta_{s}$	20’000/32	1.4% air	1.6 °C
$\theta_{s}$	100’000/32	0.22% air	0.27 °C
$\theta_{n}$	20’000/32	0.13% air	0.24 °C

**Table 2:** Summary of the values of $\bar{AE}$ for the cases shown in Figure 6A,B.

Input	Epochs/Batch Size	${\bar{\mathrm{AE}}}_{\left[O_{2}\right]}$	${\bar{\mathrm{AE}}}_{T}$
$\theta_{s}$	100’000/32	0.95% air	2.1 °C
$\theta_{n}$	20’000/32	0.87% air	1.7 °C

## Abbreviations

The following abbreviations are used in this manuscript:

SV	Stern–Volmer
Adam	Adaptive Moment Estimation
NNM	Neural Network Model
MAE	Mean Absolute Error
AE	Absolute Error
KDE	Kernel Density Estimate
ELA	Error Limited Accuracy

## References

[1] Stich M.I., Fischer L.H., Wolfbeis O.S. (2010). Multiple fluorescent chemical sensing and imaging. Chem. Soc. Rev..

[2] Borisov S.M., Seifner R., Klimant I. (2011). A novel planar optical sensor for simultaneous monitoring of oxygen, carbon dioxide, pH and temperature. Anal. Bioanal. Chem..

[3] Kameya T., Matsuda Y., Egami Y., Yamaguchi H., Niimi T. (2014). Dual luminescent arrays sensor fabricated by inkjet-printing of pressure-and temperature-sensitive paints. Sens. Actuators B Chem..

[4] Wang X.D., Wolfbeis O.S. (2014). Optical methods for sensing and imaging oxygen: Materials, spectroscopies and applications. Chem. Soc. Rev..

[5] Santoro S., Moro A., Portugal C., Crespo J., Coelhoso I., Lima J. (2016). Development of oxygen and temperature sensitive membranes using molecular probes as ratiometric sensor. J. Membr. Sci..

[6] Biring S., Sadhu A.S., Deb M. (2019). An Effective Optical Dual Gas Sensor for Simultaneous Detection of Oxygen and Ammonia. Sensors.

[7] Wolfbeis O.S. (1991). Feasibility of optically sensing two parameters simultaneously using one indicator. Chemical, Biochemical, and Environmental Fiber Sensors II.

[8] Zieger S.E., Steinegger A., Klimant I., Borisov S.M. (2020). TADF-Emitting Zn (II)-Benzoporphyrin: An indicator for simultaneous sensing of oxygen and temperature. ACS Sens..

[9] Ohira S.I., Dasgupta P.K., Schug K.A. (2009). Fiber optic sensor for simultaneous determination of atmospheric nitrogen dioxide, ozone, and relative humidity. Anal. Chem..

[10] Collier B.B., McShane M.J. (2013). Time-resolved measurements of luminescence. J. Lumin..

[11] Stehning C., Holst G.A. (2004). Addressing multiple indicators on a single optical fiber-digital signal processing approach for temperature compensated oxygen sensing. IEEE Sens. J..

[12] Jorge P., Maule C., Silva A., Benrashid R., Santos J., Farahi F. (2008). Dual sensing of oxygen and temperature using quantum dots and a ruthenium complex. Anal. Chim. Acta.

[13] Moore J.P., Higgins C., McGaughey O., Lawless B.G., MacCraith B.D. (2006). Exploiting sensor cross sensitivity: Achieving temperature compensation via a dual-element optical oxygen sensor. Advanced Environmental, Chemical, and Biological Sensing Technologies IV.

[14] Papkovsky D.B., Dmitriev R.I. (2013). Biological detection by optical oxygen sensing. Chem. Soc. Rev..

[15] Lakowicz J.R. (2006). Principles of Fluorescence Spectroscopy.

[16] Li F., Wei Y., Chen Y., Li D., Zhang X. (2015). An intelligent optical dissolved oxygen measurement method based on a fluorescent quenching mechanism. Sensors.

[17] Xu W., McDonough R.C., Langsdorf B., Demas J., DeGraff B. (1994). Oxygen sensors based on luminescence quenching: Interactions of metal complexes with the polymer supports. Anal. Chem..

[18] Draxler S., Lippitsch M.E., Klimant I., Kraus H., Wolfbeis O.S. (1995). Effects of polymer matrixes on the time-resolved luminescence of a ruthenium complex quenched by oxygen. J. Phys. Chem..

[19] Hartmann P., Trettnak W. (1996). Effects of polymer matrices on calibration functions of luminescent oxygen sensors based on porphyrin ketone complexes. Anal. Chem..

[20] Mills A. (1998). Controlling the sensitivity of optical oxygen sensors. Sens. Actuators B Chem..

[21] Badocco D., Mondin A., Pastore P., Voltolina S., Gross S. (2008). Dependence of calibration sensitivity of a polysulfone/Ru (II)-Tris (4, 7-diphenyl-1, 10-phenanthroline)-based oxygen optical sensor on its structural parameters. Anal. Chim. Acta.

[22] Dini F., Martinelli E., Paolesse R., Filippini D., D’Amico A., Lundström I., Di Natale C. (2011). Polymer matrices effects on the sensitivity and the selectivity of optical chemical sensors. Sens. Actuators B Chem..

[23] Argyriou A., Evgeniou T., Pontil M. Multi-task feature learning. Proceedings of the 19th International Conference on Neural Information Processing Systems (NIPS’06).

[24] Thrun S. Is learning the n-th thing any easier than learning the first?. Proceedings of the 8th International Conference on Neural Information Processing Systems (NIPS’95).

[25] Caruana R. (1997). Multitask learning. Mach. Learn..

[26] Zhang Y., Yang Q. (2017). A survey on multi-task learning. arXiv.

[27] Baxter J. (2000). A model of inductive bias learning. J. Artif. Intell. Res..

[28] Thung K.H., Wee C.Y. (2018). A brief review on multi-task learning. Multimed. Tools Appl..

[29] Michelucci U., Venturini F. (2019). Multi-task learning for multi-dimensional regression: Application to luminescence sensing. Appl. Sci..

[30] Carraway E., Demas J., DeGraff B., Bacon J. (1991). Photophysics and photochemistry of oxygen sensors based on luminescent transition-metal complexes. Anal. Chem..

[31] Demas J.N., DeGraff B., Xu W. (1995). Modeling of luminescence quenching-based sensors: Comparison of multisite and nonlinear gas solubility models. Anal. Chem..

[32] Michelucci U., Baumgartner M., Venturini F. (2019). Optical oxygen sensing with artificial intelligence. Sensors.

[33] Ogurtsov V.I., Papkovsky D.B. (2006). Modelling of phase-fluorometric oxygen sensors: Consideration of temperature effects and operational requirements. Sens. Actuators B Chem..

[34] Lo Y.L., Chu C.S., Yur J.P., Chang Y.C. (2008). Temperature compensation of fluorescence intensity-based fiber-optic oxygen sensors using modified Stern–Volmer model. Sens. Actuators B Chem..

[35] Zaitsev N., Melnikov P., Alferov V., Kopytin A., German K. (2016). Stable optical oxygen sensing material based on perfluorinated polymer and fluorinated platinum (II) and palladium (II) porphyrins. Procedia Eng..

[36] Venturini F., Michelucci U., Baumgartner M., Berghmans F., Mignani A.G. (2020). Dual oxygen and temperature sensing with single indicator using multi-task-learning neural networks. Optical Sensing and Detection VI.

[37] Michelucci U. (2018). Applied Deep Learning—A Case-Based Approach to Understanding Deep Neural Networks.

[38] Kingma D.P., Ba J.A. Adam: A method for stochastic optimization. Proceedings of the 3rd International Conference on Learning Representations, ICLR 2015.

